# Cognitive Reserve Moderates Effects of White Matter Hyperintensity on Depressive Symptoms and Cognitive Function in Late-Life Depression

**DOI:** 10.3389/fpsyt.2020.00249

**Published:** 2020-04-08

**Authors:** Chemin Lin, Chih-Mao Huang, Yang-Teng Fan, Ho-Ling Liu, Yao-Liang Chen, Howard J. Aizenstein, Tatia Mei-Chun Lee, Shwu-Hua Lee

**Affiliations:** ^1^ Department of Psychiatry, Keelung Chang Gung Memorial Hospital, Keelung, Taiwan; ^2^ College of Medicine, Chang Gung University, Taoyuan County, Taiwan; ^3^ Community Medicine Research Center, Chang Gung Memorial Hospital, Keelung, Taiwan; ^4^ Department of Biological Science and Technology, National Chiao Tung University, Hsinchu, Taiwan; ^5^ Center for Intelligent Drug Systems and Smart Bio-devices (IDS2B), National Chiao Tung University, Taipei, Taiwan; ^6^ Department of Imaging Physics, University of Texas MD Anderson Cancer Center, Houston, TX, United States; ^7^ Department of Medical Imaging and Intervention, Chang Gung Memorial Hospital, Keelung, Taiwan; ^8^ Department of Psychiatry, University of Pittsburgh, Pittsburgh, PA, United States; ^9^ Department of Bioengineering, University of Pittsburgh, Pittsburgh, PA, United States; ^10^ Laboratory of Neuropsychology, The University of Hong Kong, Hong Kong, Hong Kong; ^11^ State Key Laboratory of Brain and Cognitive Science, The University of Hong Kong, Hong Kong, Hong Kong; ^12^ Department of Psychiatry, Linkou Chang Gung Memorial Hospital, Taoyuan County, Taiwan

**Keywords:** cognitive reserve, education, white matter hyperintensity, late-life depression, verbal fluency, cognitive function

## Abstract

**Introduction:**

White matter hyperintensity (WMH) has been regarded as one of the major contributor of the vascular hypothesis of late-life depression (LLD) and cognitive decline in the elderly. On the other hand, cognitive reserve (CR) has long been hypothesized to provide resilience and adaptability against age- and disease-related insults. This study examined the role of CR, using proxy of education, in moderating the association between WMH and clinical LLD expression.

**Methods:**

A total of 54 elderly diagnosed with major depressive disorder and 38 matched healthy controls participated in this study. They received MRI scanning and a battery of neuropsychological tests. WMH was quantified by an automated segmentation algorithm. Linear regression analyses were conducted separately in the LLD and control groups to examine the effects of WMH, education and their interaction in depression severity and various cognitive domains.

**Results:**

WMH was significantly and negatively associated with executive function only in the healthy controls. In patients with LLD, we observed a significant interactive effect in education on the association between WMH and depression severity and language domain (category fluency task). Specifically, those with high education showed less depressive symptoms and cognitive decline as WMH increased.

**Conclusion:**

WMH is associated with lower cognitive function. However, in patients with LLD, high education attenuates the deleterious effect of WMH on mood and cognition. Therefore, CR appears to exert a protective effect on neurocognitive functioning in people with LLD.

## Introduction

Late-life depression (LLD) is a common psychiatric disorder associated with disability, decreased mental well-being, and completed suicides in the elderly (1). On the contrary to mildlife depression, LLD is substantially attributed to aging process and cerebrovascular changes (2). Such characteristics have given rise to the formation of the “vascular depression” hypothesis, which states that cerebrovascular factors predispose, precipitate, or perpetuate the geriatric depressive syndromes (3). Since the advent of magnetic resonance imaging (MRI), neuroimaging-defined vascular changes, particularly the white matter hyperintensities (WMH), have provided ample evidence in support of the vascular hypothesis of LLD (2, 4).

Pathologically, WMH is caused by demyelination, gliosis, and axonal loss in the periventricular or deep white matter (5). Clinically, WMH occurs with normal aging, and is associated with increased risk of subsequent stroke, dementia, and death (6). Importantly, a fourth-fold increase in the prevalence of having WMH was found in late-onset compared with early-onset LLD (7). It is hypothesized that WMH strategically disrupts the communication between cortical and subcortical regions, causing the frontolimbic compromise, and gives rise to affective and cognitive symptoms in LLD (2). Frontolimbic dysfunction was evidenced by the heightened limbic affective reactivity in depressed elderly patients with high WMH loading (8). Moreover, WMH was associated with cognitive impairment in LLD (9). Although WMH could predict poor antidepressant response in LLD, its predictive power was out-performed by the baseline cognitive function (10). However, some studies have failed to find an association between high WMH and poor antidepressant response (11, 12). Therefore, other factors should be considered when assessing the impact of WMH on depression and cognitive function in LLD.

Cognitive reserve (CR) is the notion encompassing the active process of efficient utilization of brain networks in an effort to sustain normal functions despite brain insults (13, 14). Indicated by education, occupational attainment, or leisure activities, CR could reduce or delay the occurrence of dementia (15). Similarly, CR may reduce cognitive decline in LLD by ameliorating the detrimental effects incurred by cerebrovascular diseases and hippocampal atrophy (16). In a large population-based cohort, CR could moderate the negative association between depression and cognitive function (17). However, a previous study reported that high education did not buffer the deleterious effect of LLD on cognitive decline (18). Furthermore, in another community-based sample, those with high CR showed more pronounced cognitive decline as depressive symptoms escalated (19).

Previous studies in the literature suggest that both CR and WMH are indispensable factors and should be considered together in the evaluation of the depressive symptoms and cognitive function in the elderly. A few studies have been conducted in normal elderly individuals have shown that education may modify the negative association between WMH and processing speed (20) or other cognitive domains (21). Similarly, cognitive leisure activities may buffer the negative association between WMH and processing speed (22). However, parallel studies in LLD are scarce. Therefore, based on the framework of the vascular hypothesis in LLD, we specifically examined whether CR (using the proxy of education) could modify effects of WMH on depressive symptoms and different cognitive domains. Based on the prior findings, we hypothesized that WMH will be associated with more severe depressive symptoms and cognitive decline in elderly individuals with lower CR than in those with higher CR.

## Methods

### Participants

Following the approval of the institutional review board from Chang Gung Memorial Hospital (IRB number: IRB104-0928C), we recruited elderly patients from the psychiatric department of Chang Gung Memorial Hospital. All of them had been informed about the purpose of the study with written consent. Patients were at least 60 years of age, with their first major depressive episode (MDE) occurring after 50 years of age (i.e., late onset). Diagnostic interviews were conducted by certified geriatric psychiatrists (C. Lin and S.W. Lee) based on the DSM-5 criteria were conducted to ascertained the MDE diagnosis. Patients with other major mental illnesses, including psychotic disorders, bipolar disorder, substance use disorders, and major neurocognitive disorders were excluded. However, patients with anxiety disorders were included owing to high comorbidity with depression. Elderly controls recruited *via* advertisement presented no life-time history of Axis I major mental disorders. All participants were right-handed and scored at least 24 on the Mini-Mental State Examination (MMSE). Other exclusion criteria for both groups included a history of significant head trauma (with loss of consciousness), major neurological disorder, stroke, thyroid dysfunction, or other systemic illnesses. Due to ethical considerations, a steady dose of psychotropics was maintained in LLD patients for at least 2 months before MRI.

### Behavioral Measures

The following assessments were performed on the day of functional MRI (fMRI): 15-item Geriatric Depressive Scale (GDS) (23) and an array of neuropsychological tests including digit symbol substitution test (DSST, where participants need to write down the symbols below an array of numbers as fast as they can based on the pairing rules in the instruction), digit span forward/backward (i.e. to test the longest sequence one can remember in a normal or reverse order after presented with the sequence), letter-number sequencing (LNS, where participants must respectively recite the letters and numbers in an alphabetic and ascending order after given a group of random letters and numbers) (24), facial memory (memory domain; where participants are presented with 24 faces, 1 at a time for 2 seconds, and then are asked to identify these faces among 48 faces, 24 seen and 24 unseen), and category verbal fluency (language domain; where participants need to name as many words as possible in 60 s in the category of colors, animals, fruits, and towns without repeating). We derived the other two cognitive domains—processing speed and working memory—by averaging the scores of the DSST plus digit span forward and digit span backward plus LNS. All scores of the neuropsychological tests were standardized by using the mean and standard deviation of scores of the normal controls. The z-score of the DSST was reversed such that higher values in each domain represented higher functioning. Education (in years) was selected a proxy for CR in this study.

### MRI Acquisition and Image Preprocessing

MRI data were collected using an 8-channel head coil on a 3T MRI scanner (Discovery MR750, GE Healthcare, Milwaukee, WI). T1-weighted structural images were acquired as follows: TR = 8 ms, TE = 3 ms, flip angle = 12°, FOV = 250 × 250 mm^2^, voxel size = 0.98 × 0.98 × 1 mm^3^, slice number = 160. Moreover, T2-weighted FLAIR scans were acquired (TR/TE= 9,000 ms/140ms, inversion time = 2,250 ms, matrix = 320 × 224, slice thickness = 3.5 mm, slice number = 32) with a 0.5-mm gap. A semi-automated segmentation procedure was followed to derive total brain volume and WMH volume, as previously described (25). Briefly, WMH was quantified using the fuzzy connectedness segmentation algorithm on WM lesions from T2-FLAIR images. WMH was registered and localized onto the John Hopkins University White Matter Atlas. WMH was subsequently divided by total brain volume and log-transformed to generate the normalized WMH, serving as a marker for cerebral vascular burden for linear regression analysis.

### Statistical Analysis

We first compared the group differences in demographic and behavioral data as well as WMH loading between patients of LLD and normal controls. We then performed serial linear regressions using GDS and the four cognitive domains as dependent variables; education, WMH, and the interaction between education and WMH were independent variables, with age and sex as covariates of no interest. Education and WMH were centered with their means before creating the interaction variable to avoid multi-collinearity. These five independent regressions were repeated in the LLD and control groups separately. All analyses above were performed using SPSS v21 (SPSS, Inc., Chicago, IL, USA), with significance level set at *p* < 0.05.

## Results

We enrolled 54 patients with LLD and 38 normal controls. The first MDE occurred at an average age of 61.0 ± 6.1 years with a mean of 1.5 ± 1.1 episodes. The average patient age in the LLD group was 66.8 ± 5.6 years, and that in the control group was 68.2 ± 5.3 years. The average duration of education was 7.3 ± 2.5 years in the LLD group, which was significantly lower than 11.2 ± 4.3 years in the control group. As expected, the LLD group scored higher on GDS than the control group (7.3 ± 2.5 versus 2.6 ± 2.0, respectively). Furthermore, LLD patients showed marginally lower MMSE scores and significantly lower neurocognitive function in terms of processing speed, working memory, and language domain. However, no significant group difference was found in WMH load and memory function (**Table 1**). In terms of medical comorbidities, 19 out of all 92 pariticipants were diagnosed of hypertension (20.7%), 9 (9.8%) of hyperlipidemia, 7 (7.6%) of diabetes mellitus, 9 (9.8%) of hyperlipidemia, 2 (2.2%) of coronary artery disease, and 1 (1.1%) of osteoarthritis.

**Table 1:** Demographic and clinical features of patients with late-life depression and elderly controls.

	LLD	NC	Statistics
	(n = 54)	(n = 38)	
Age	66.8 ± 5.6	68.2 ± 5.3	t=-1.21, p=0.230
Gender (M/F)	20/35	14/24	χ2 = 0.01, p=0.990
Education	7.3 ± 2.5***	11.2 ± 4.3	t=-5.01, p < .001
Geriatric Depression Scale	7.6 ± 3.5***	2.6 ± 2.0	t=8.62, p < .001
MMSE	27.4 ± 2.4	28.2 ± 1.5	t=-1.92, p=0.060
Log normalized WMH	-3.7 ± 0.5	-3.9 ± 0.6	t=1.21, p=0.260
Neurocognitive function			
Processing Speed	-0.755 ± 0.779***	0 ± 0.752	t=-4.65, p < 0.001
Working Memory	-0.455 ± 0.748*	-0.032 ± 0.96	t=-2.34, p=0.021
Memory	-0.028 ± 0.943	-0.021 ± 1.011	t=-0.03, p < 0.973
Language	-0.772 ± 0.783***	0.004 ± 1.02	t=-4.12, p < 0.001

*P < 0.05; ***P < 0.001; Mini-Mental State Examination, MMSE; Values in the neurocognitive function are z-scored.

**Figure 1:**
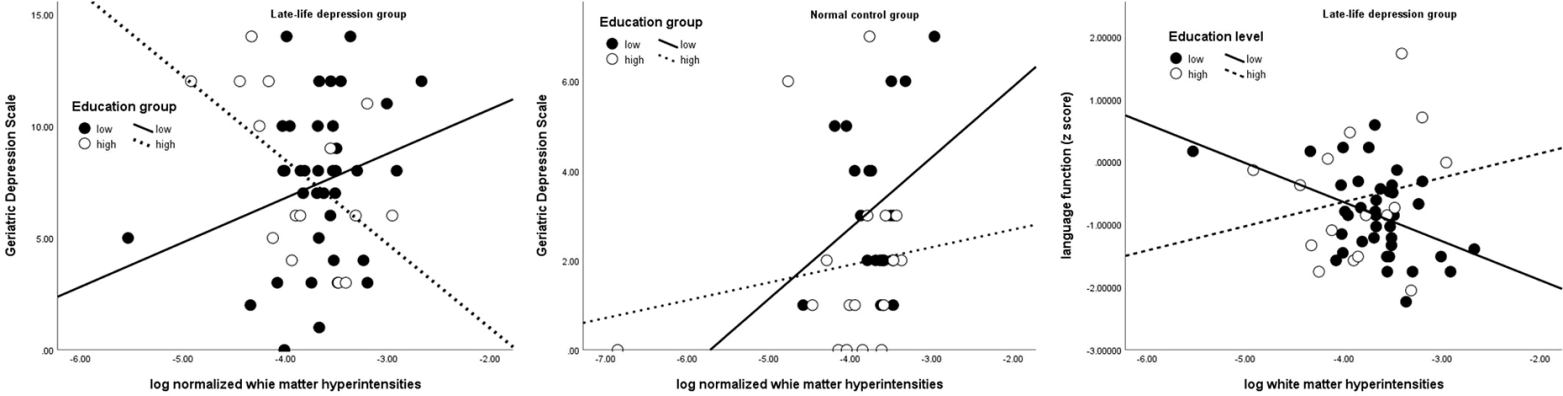
Interactive effect of education on the association between white matter hyperintensity, depressive severity (left and middle), and language domain (right) Solid line and black dots represent low education group; dotted line and white dots represent high education group.

Next, we tested whether education could moderate effects of WMH on depressive symptoms and cognitive function. In LLD patients, there was no main effect of education or WMH alone on GDS, but there was a significant interaction effect of these parameters on GDS (β = −0.96, 95% CI = −1.87, −0.04; *p* = 0.040). This effect remained significant after controlling for antidepressant loading (*p* = 0.045) or MMSE scores (β = −0.97). Furthermore, more depressive symptoms were associated with higher WMH only in individuals with low education (**Figure 1**). No main or interaction effect was found in controls.

Education was positively associated with processing speed both in the LLD (β = 0.13, 95% CI = 0.05, 0.21; *p* = 0.002) and control groups (β = 0.08, 95% CI = 0.01, 0.14; *p* = 0.018) and with language only in the control group (β = 0.12, 95% CI = 0.04, 0.20; *p* = 0.003). WMH was negatively associated with working memory (β = −1.38, 95% CI = −2.36, −0.40; *p* = 0.007) in the control group. In the language domain, an interaction effect between education and WMH was noted only in LLD patients (β = 0.23, 95% CI = 0.02, 0.44; *p* = 0.030) (**Table 2**), wherein those with high education and high WMH loads showed less decline in language function than those with low education (**Figure 1**). This interaction effect remained significant even when GDS or antidepressant loading was entered into the regression (β = 0.23, *p* = 0.041).

**Table 2:** Linear regression showing the association of white matter hyperintensity (WMH), education, and their interaction with depressive symptoms and different cognitive domains.

Group	Variables	unstandardized β [95% CI]									
		GDS		Processing speed		Working memory		Language		Memory	
LLD	Intercept	4.97	[-12.35, 22.28]	-0.78	[-4.34, 2.77]	1.46	[-2.26, 5.18]	0.62	[-2.72, 6.72]	2.00	[-2.72, 6.72]
	Age	0.02	[-0.16, 0.20]	-0.02	[-0.06, 0.01]	-0.03	[-0.07, 0.00]	-0.02	[-0.10, 0.00]	-0.05	[-0.10, 0.00]
	Gender	-2.00	[-4.02, 0.01]	0.15	[-0.26, 0.57]	-0.12	[-0.55, 0.31]	0.01	[-0.45, 0.65]	0.10	[-0.45, 0.65]
	Education	-0.10	[-0.52, 0.31]	0.13**	[0.05, 0.21]	0.06	[-0.03, 0.14]	0.05	[-0.07, 0.15]	0.04	[-0.07, 0.15]
	WMH	-1.32	[-3.8, 1.16]	-0.10	[-0.61, 0.41]	-0.05	[-0.58, 0.48]	0.11	[-0.90, 0.45]	-0.22	[-0.09, 0.45]
	WMH x Education	-0.96*	[-1.87, -0.04]	0.10	[-0.09, 0.29]	0.11	[-0.09, 0.30]	0.23*	[0.02, 0.44]	0.05	[-0.20, 0.30]
NC	Intercept	-3.93	[-17.82, 9.96]	1.83	[-3.45, 7.12]	-3.25	[-9.63, 3.12]	-0.52	[-7.20, 6.15]	2.28	[-5.36, 9.93]
	Age	0.13	[-0.01, 0.26]	-0.05	[-0.1, 0.01]	-0.03	[-0.10, 0.03]	-0.04	[-0.11, 0.02]	-0.03	[-0.10, 0.05]
	Gender	0.71	[-0.64, 2.05]	0.39	[-0.12, 0.91]	-0.13	[-0.74, 0.49]	0.24	[-0.40, 0.89]	0.42	[-0.32, 1.16]
	Education	-0.24	[-0.40, -0.07]	0.08*	[0.01, 0.14]	0.05	[-0.02, 0.13]	0.12**	[0.04, 0.20]	0.07	[-0.02, 0.16]
	WMH	0.17	[-1.97, 2.30]	0.07	[-0.74, 0.88]	-1.38*	[-2.36, -0.40]	-0.46	[-1.49, 0.56]	0.52	[-0.65, 1.70]
	WMH x Education	0.09	[-0.25, 0.43]	-0.05	[-0.18, 0.08]	0.14	[-0.02, 0.30]	0.00	[-0.17, 0.16]	-0.14	[-0.33, 0.05]

LLD, Late-life depression; NC, normal control; p < 0.05*; p < 0.005**

Lastly, given the interactive effect of education and WMH on GDS and language function only in LLD group, we tested whether if there was a three-way interaction between group, education and WMH across all subjects. A three-way interaction was found in group x education x WMH in predicting GDS (β = −1.08, 95% CI = −1.92, −0.23; *p* = 0.013), but not in predicting language function (β = 0.20, 95% CI = 0.12, −0.03; *p* = 0.096) (**Table 3** and **Figure 1**).

**Table 3:** Three-way interaction effect between group, education, and white matter hyperintensity (WMH) on the depressive symptoms and cognitive function in language domain across all the participants.

Variables	unstandardized β [95% CI]			
	GDS		Language	
intercept	2.85	[-24.93,30.62]	-1.74	[3.95,-9.48]
Age	0.05	[-0.06,0.17]	-0.03	[0.02,-0.06]
Gender	0.93	[-0.3,2.15]	-0.11	[0.17,-0.45]
Education	-0.26	[-1.99,1.46]	0.15	[0.25,-0.33]
Group	30.82	[-1.44,63.08]	-4.12	[4.58,-13.10]
WMH	0.38	[-6.44,7.21]	-0.65	[0.97,-2.56]
Group x Education	-3.90*	[-7.17,-0.63]	0.70	[0.46,-0.21]
Group x WMH	7.47	[-1.13,16.07]	-1.14	[1.22,-3.54]
Education x WMH	0.00	[-0.47,0.46]	0.01	[0.07,-0.12]
Group x Education x WMH	-1.08*	[-1.92,-0.23]	0.20	[0.12,-0.03]

WMH, White matter hyperintensities; GDS, geriatric depression scale; p < 0.05*.

## Discussion

Our findings demonstrate that the effects of WMH on depressive symptoms and cognitive function in LLD depend on education level. Higher education may mitigate the negative association of WMH with depressive symptoms and language function. This implies that high CR, using the proxy of education level, could moderate deleterious effects of WMH in LLD.

Severe WMH is associated with higher prevalence of depressive symptoms (26). In late-onset LLD, higher WMH in the left superior longitudinal fasciculus and the right uncinate fasciculus was positively correlated with depression severity (27). As most of our patients were under antidepressant treatment for a period of time, higher depressive symptoms in current study may be indicative of greater refractoriness or treatment resistance. Similarly, recent evidence has demonstrated that non-remitters showed significantly WMH increase than remitters following a 12-week antidepressant course against LLD (28). Our results further extend these findings by showing such an association only in those with low education. A number of epidemiological studies have proposed lower education as a risk factor for LLD (29–31), with socioeconomic disparity as the probable cause for this association. However, uncovering the neuroscientific basis of this phenomenon requires application of the framework of CR to the existing vascular hypothesis of LLD.

Lower WM integrity on diffusion tensor imaging was found in elderly patients with high CR, measured in terms of education level (32) or life-long bilingualism (33); those with high CR were speculated to be better able to endure WM lesions. The specific WM tracts related to CR include the inferior longitudinal fasciculus/inferior fronto-occipital fasciculus, the fornix (33), and corpus callosum (32, 33). However, these do not coincide with the tracts (e.g., cingulum bundle, uncinated fasciculus, and superior longitudinal fasciculus) typically reported to harbor WMH in LLD (34, 35). Given this discrepancy, education may enhance network efficiency and re-organization of the network topology (36), circumventing the tracts with high WMH in order to maintain function. For example, the corpus callosum, which is involved in CR, is crucial for inter-hemispheric communication (37), supporting the claim that CR promotes brain plasticity in the face of brain pathology (38).

In this study, we found WMH to be associated with lower working memory in elderly controls. This is consistent with other studies showing that lower performance of WM was associated with high WMH in the periventricular region (39, 40). Furthermore, we found that education modified effects of WMH on language function in LLD patients. Here, we measured the language function by using categorical verbal fluency—a parameter that is amenable to effects of education (41). Although working memory is required as verbal fluency is a speeded task (42, 43), language function is the most critical component in this task (44). Multiple brain areas, such as the left inferior/middle frontal gyrus, anterior cingulate gyrus (ACC) (45), left superior parietal lobule and left hippocampal formation (46), are activated in the verbal fluency task. Conversely, hypoactivation in the left prefrontal cortex, left ACC and frontopolar region is associated with poor task performance in depression (47) or late-onset LLD (48). Since WMH disrupts long range connections in the brain, it affects executive function or working memory is affected (49) and jeopardizes verbal fluency function. As opposed to the age decline in fluid intelligence (Gf), crystallized intelligence (Gc) is relatively spared (50, 51). Benefits bestowed by education on Gc may offset detrimental effects of WMH on Gf, thereby sustaining the integrity of verbal fluency. However, how education attainment preserves language functioning is out of the scope of the current study. A possible explanation is that it promotes synaptogenesis, which in turn increases the redundancy and neuroplasticity of the brain (52).

It is intriguing that CR moderates the effects of WMH on mood and cognitive function in a much more significant way in LLD patients than in normal elderly individuals. For example, a three-way interaction between group, education and WMH was found in predicting the scores of GDS. A possible explanation lies in the threshold theory (53, 54), which posits that cognitive function is compromised only after the WMH load reaches a certain threshold. Therefore, low WMH load in the elderly controls may be insufficient to observe benefits of CR. Alternatively, a brain with LLD may be functionally and structurally more compromised, rendering those afflicted with depression more susceptible to other insults. Since overall WMH in our LLD patients did not surpass that in the elderly controls, we suspect that these lesions may be located in strategic areas that control cognitive and emotional function. Thus, CR could play a pivotal role in linking these symptoms to LLD (16). This may explain the failure of a previous study in demonstrating a significant moderating effect of CR on the association between depressive symptoms and WMH as it could be due to inclusion of participants not formally diagnosed with depression (19).

### Limitations and Conclusions

Our study has a few limitations. First, due to ethical concerns, all the participants were receiving pharmacotherapy at the time of data collection. However, this does not influence our interaction results since the antidepressant dose did not differ between high or low education individuals within the LLD group. Second, we used education as a proxy for CR. Further studies incorporating occupational attainment and leisure activities may provide a more comprehensive evaluation of CR. Third, we did not specify periventricular or deep WMH, which may result in a lack of association in other cognitive domains. However, periventricular and deep WMH are highly correlated and may all reflect total WMH (55). Moreover, we specifically excluded patients with stroke or significant cardiovascular comorbidity, resulting in relatively low WMH loads in both groups. Therefore, we evaluated total WMH in order to gain more power in the analysis. Finally, our study enrolled a small sample size and only examined WMH. Studies with larger sample size evaluating other biomarkers for cognitive decline (e.g., amyloid deposition or tau pathology) are warranted to further elucidate the underlying mechanism.

In conclusion, we demonstrated that education is an important modifier of the association between WMH and clinical LLD presentation. Our finding implies that education level must be taken into consideration while analyzing consequences of WMH in LLD. Previous discrepancies in LLD research may be attributed to the failure to consider CR and WMH at the same time. Overall, our result reiterates the notion that education is related to increased CR and supports the hypothesis that higher CR confers a higher threshold for symptom manifestation of LLD (16, 56).

## Data Availability Statement

The datasets generated for this study are available on request to the corresponding authors.

## Ethics Statement

The institutional review board of the Chang Gung Memorial Hospital approved this study (IRB number:IRB104-0928).

## Author Contributions

CL, C-MH, H-LL, S-HL, and TL designed the project. CL, H-LL, Y-LC, S-HL, and TL performed the experiment. CL, C-MH, Y-TF, and HA analyzed the data. CL wrote the manuscript. All authors have read and approved the final version of the manuscript.

## Funding

This work was supported by (1) medical research grants CMRPG2J0351 and CRRPG2G0061/62 from Chang Gung Memorial Hospital and NRRPG2H0031, NMRPG3G6031/32 from Ministry of Science and Technology, Taiwan to CL, (2) the KKHo International Charitable Foundation to TL and (3) this work was also partially supported by Taiwan’s Ministry of Science and Technology (107-2410-H-009-028 -MY3; 108-2321-B-038-005-MY2) and by the Center for Intelligent Drug Systems and Smart Bio-devices (IDS2B) from The Featured Areas Research Center Program within the framework of the Higher Education Sprout Project by the Ministry of Education(MOE) in Taiwan to CMH.

## Conflict of Interest 

The authors declare that the research was conducted in the absence of any commercial or financial relationships that could be construed as a potential conflict of interest.

The handling editor declared a past co-authorship with one of the authors, HA.
